# Impact of killer-immunoglobulin-like receptor and human leukocyte antigen genotypes on the efficacy of immunotherapy in acute myeloid leukemia

**DOI:** 10.1038/leu.2017.151

**Published:** 2017-06-23

**Authors:** E Bernson, A Hallner, F E Sander, O Wilsson, O Werlenius, A Rydström, R Kiffin, M Brune, R Foà, J Aurelius, A Martner, K Hellstrand, F B Thorén

**Affiliations:** 1 TIMM Laboratory, Sahlgrenska Cancer Center, University of Gothenburg, Gothenburg, Sweden; 2 Department of Hematology, University of Gothenburg, Gothenburg, Sweden; 3 Department of Cellular Biotechnologies and Hematology, Sapienza University, Rome, Italy

## Abstract

Interactions between killer-immunoglobulin-like receptors (KIRs) and their HLA class I ligands are instrumental in natural killer (NK) cell regulation and protect normal tissue from NK cell attack. Human KIR haplotypes comprise genes encoding mainly inhibitory receptors (KIR A) or activating and inhibitory receptors (KIR B). A substantial fraction of humans lack ligands for inhibitory KIRs (iKIRs), that is, a ‘missing ligand’ genotype. KIR B/x and missing ligand genotypes may thus give rise to potentially autoreactive, unlicensed NK cells. Little is known regarding the impact of such genotypes in untransplanted acute myeloid leukemia (AML). For this study, NK cell phenotypes and KIR/HLA genotypes were determined in 81 AML patients who received immunotherapy with histamine dihydrochloride and low-dose IL-2 for relapse prevention (NCT01347996). We observed that presence of unlicensed NK cells impacted favorably on clinical outcome, in particular among patients harboring functional NK cells reflected by high expression of the natural cytotoxicity receptor (NCR) NKp46. Genotype analyses suggested that the clinical benefit of high NCR expression was restricted to patients with a missing ligand genotype and/or a KIR B/x genotype. These data imply that functional NK cells are significant anti-leukemic effector cells in patients with KIR/HLA genotypes that favor NK cell autoreactivity.

## Introduction

Acute myeloid leukemia (AML) is a genetically and morphologically heterogeneous disease characterized by the expansion and accumulation of immature myeloid cells in the bone marrow and peripheral blood. The prognosis is determined by risk factors such as chromosomal abnormalities, gene mutations and age, and based on these and other factors AML can be classified into high-, intermediate- or low-risk disease. Depending on the risk category, between 65 to as low as 5% of patients experience long-term survival.^1^ Despite achieving complete remission (CR) in response to chemotherapy, alone or combined with autologous stem cell transplantation, the vast majority of intermediate and high-risk AML patients are not cured as a small residual clone of leukemic cells may expand to cause relapse with poor prospects of long-term survival.^2^ To prevent relapse, younger patients may receive an allogeneic stem cell transplant (allo-SCT), but not all patients are eligible for transplantation and additional strategies to avoid relapse in non-transplanted patients are highly warranted.^3^

Numerous studies of allo-transplanted and non-transplanted patients have highlighted the importance of cellular immunity, including aspects of natural killer (NK) cell function, for the outcome of AML.^4, 5, 6^ In order to identify aberrant cells, NK cells rely on the surface expression of a set of activating receptors such as natural cytotoxicity receptors (NCRs), including NKp46 and NKp30. The activating signals conveyed by these receptors can be prevented by inhibitory NK cell receptors, mainly inhibitory KIRs (iKIRs) and CD94-NKG2A that target class I HLA antigens.^7, 8^ The KIR ligands are divided into three major groups based on the amino acid in positions 77 and 80 in the KIR-binding domain. Thus, HLA-C alleles belong either to the C1 group (recognized by KIR2DL2 or KIR2DL3) or the C2 group (recognized by KIR2DL1), while HLA-B or HLA-A alleles may contain a Bw4 motif that is recognized by KIR3DL1.^9^ The KIRs are encoded by genes in the highly polymorphic KIR locus. There are two main KIR haplotypes; the A haplotype that comprises genes for iKIRs and KIR2DS4, and the B haplotype that in addition to *iKIR* genes also carries genes for a variable set of up to 6 activating KIRs (aKIRs).^10, 11, 12^ The ligands for aKIRs are not completely characterized, but KIR2DS1 recognizes HLA-C2, and KIR2DS2 recognizes HLA-A11 and HLA-C1.^13, 14, 15, 16, 17^

Under normal conditions, the functional competence of individual NK cells is set by their steady-state input of inhibitory and activating signals in a process known as NK cell education.^18, 19, 20^ As *KIR* genes and HLA genes are located on different chromosomes, they are inherited independently and many individuals thus have a genotypic discordance between HLA alleles and *KIR* genes with a lack of inhibitory ligands (‘missing ligand genotype’).^9, 21^ As KIR expression is stochastic in NK cells, a missing ligand genotype entails substantial numbers of NK cells that only express inhibitory receptors for non-self HLA (hereafter referred to as NS-iKIR NK cells).^18^ These potentially autoreactive NS-iKIR NK cells, or unlicensed NK cells, do not receive inhibitory input and will remain hyporesponsive to target cells under steady-state conditions.^19, 20^ Conversely, NK cells that engage in interactions between activating KIRs (S-aKIRs) and corresponding HLA ligands will constantly receive activating signals and become disarmed/hyporesponsive to avoid autoreactivity.^14, 22^

Recent studies suggest that perturbations of the immune homeostasis, such as autologous transplantation, treatment with monoclonal antibodies and viral infections, may activate otherwise hyporesponsive NK cells to contribute to eradication of aberrant cells.^21, 23, 24, 25, 26^ In addition, *in vitro* studies imply that proinflammatory cytokine stimulation may render unlicensed NK cells responsive.^27, 28^ In a phase III trial, relapse-preventive immunotherapy combining low-dose IL-2 and histamine dihydrochloride (HDC/IL-2) was shown to significantly improve leukemia-free survival (LFS) for AML patients.^29^ The rationale for this immunotherapy is to enhance the cytotoxic function of the lymphocytic population using the synergistic effect of IL-2 stimulation along with histamine dihydrochloride, which targets the secretion of immunosuppressive reactive oxygen species (ROS) from myeloid cells.^3, 30, 31^ In a recent phase IV trial in AML, where patients were given HDC/IL-2, this strategy was found to strongly upregulate the expression of NCRs on NK cells and induce NK cell expansion, and a high expression of NKp46 was associated with significantly superior LFS and overall survival (OS).^32, 33^

For the present study, we aimed to clarify whether KIR/HLA genotypes impact on relapse risk of AML patients receiving HDC/IL-2. Our results suggest that HDC/IL-2 immunotherapy may induce break of tolerance in patients, and that the reported benefit from intact NCR expression is restricted to patients with either a KIR B/x genotype or a missing inhibitory ligand genotype.

## Patients and methods

### Patients

The single-armed multicenter phase IV study (Re:Mission, NCT01347996) enrolled 84 patients (age 18–79) with AML in first CR who received ten 21-day cycles of HDC/IL-2 for 18 months or until relapse/death. Sample size was chosen in collaboration with regulatory authorities to meet the primary endpoints, which included the assessment of the pharmacodynamic effects of HDC/IL-2 by monitoring T and NK cell phenotypes before and after treatment cycles. Results from the trial have been published previously.^32, 33, 34, 35^ The herein reported aspects of NK cell biology vs clinical outcome (LFS) were not specified in the protocol and were performed *post* *hoc*. A detailed account for patient characteristics, such as age, mutation status and European Leukemia Net risk class,^36^ is found in previous study reports.^32, 33^ The study of pharmacodynamic effects on different effector NK cell subsets in the clinical trial were approved by the Ethics Committees of each participating institution, and all patients gave written informed consent before enrollment. Untreated patients, newly diagnosed with AML, at the Sahlgrenska University Hospital were asked to donate blood for a phenotypic and functional characterization of normal and malignant leukocytes. All patients gave written informed consent to participation in the study. Peripheral blood mononuclear cells (PBMC) from patients were isolated and cryopreserved.

### Sampling of peripheral blood and flow cytometry of Re:Mission study samples

Peripheral blood was collected before and after the first and third treatment cycles, referred to as C1D1 (Cycle1, Day1), C1D21, C3D1 and C3D21). Patients relapsing and failure of samples to meet viability criteria resulted in varying numbers of observations at different time points (NCR panel *n*=62, 62 and 53; KIR panel *n*=60, 64 and 49 for C1D1, C1D21 and C3D1, respectively). Forty-four paired observations (C1D1–C1D21) were available for the CD8 T-cell panel. Peripheral blood mononuclear cells were isolated and cryopreserved at local sites and shipped to the central laboratory at University of Gothenburg for analysis by flow cytometry. Samples with <25% viability were excluded. Differential counts of whole blood were utilized to calculate absolute counts of blood NK cell phenotypes. A full list of fluorochrome-conjugated antibodies is found in the Supplementary Material. High and low NKp46 expression was defined as above or below median NKp46 expression, as measured by the median fluorescence intensity of NKp46 on CD16^+^ NK cells.^32^ Amongst CD8^+^ T cells, effector memory T cells (T_EM_) were defined as CD45RO^+^CCR7^−^ cells, and effector T cells (T_eff_) were defined as CD45RA^+^CCR7^−^ cells.^34^

### DNA extraction and KIR/HLA genotyping

DNA was extracted from whole blood using a Roche MagNAPure 96 (Pleasanton, CA, USA) instrument according to the manufacturer’s instructions. KIR typing was performed using the One Lambda KIR SSO Genotyping Test. When the analysis generated conflicting results for certain KIR alleles, the Olerup SSP KIR genotyping kit was used to determine the KIR genotype. KIR ligands were determined using the Olerup SSP KIR HLA Ligand kit.

Patients were dichotomized based on the concordance/discordance between their KIR and HLA genotypes. Patients lacking an HLA ligand for one self-expressed inhibitory KIR (2DL1—HLA-C2; DL2/3—HLA-C1; 3DL1—HLA Bw4) were considered to have a ‘missing ligand’ genotype.

### IL-2 stimulation of PBMC and degranulation assay

Healthy donor PBMCs were thawed and stimulated overnight in 500 U/ml IL-2 (Proleukin, Novartis Pharmaceuticals, Surrey, UK). AML samples were thawed, stained for CD34^+^, and the CD34^**+**^ blasts were sorted on a 3-laser FACSARIA III flow cytometer (405, 488 and 640 nm; BD Biosciences, San Diego, CA, USA). K562 cells or sorted CD34^**+**^ AML cells were added to the cultured PBMCs at a ratio 4:1 (PBMC:target cells) and incubated for 4 h in presence of CD107a antibody. After incubation, cells were washed and stained with antibodies directed against CD3, CD56, CD107a, NKG2A, KIR2DL1/S1, KIR2DL2/L3 and KIR3DL1 followed by analysis using a BD LSR Fortessa SORP instrument.

### Statistical analyses

In accordance with the statistical plan, paired two-sided *t*-tests were used for single comparisons of NK cell phenotypes. For multiple comparisons within a dataset, one-way ANOVA followed by Bonferroni’s multiple comparison test, were used. The analyses of NK or T-cell phenotype vs outcome are based on the data for LFS as described previously.^32^ The impact on LFS was analyzed using the log-rank test. To optimize cutoff points for high and low frequencies of NKG2A^−^ NS-iKIR NK cells, receiver-operating characteristics (ROC) curves and Youden index were used,^37^ determining a cutoff value optimal for the specific analysis instead of using a median value. The area under the receiver-operating characteristic curve (AUROC) and 95% confidence interval (CI) are reported in Supplementary Table 1. Parameters that significantly predicted LFS using the log-rank test were further analyzed by univariable and multivariable Cox regression analysis. Covariates, including age, risk group classification, number of induction courses required to achieve CR (1 or >1) and number of consolidation courses (0–2 or >2) were tested in univariable analyses. Covariates with *P*-values below 0.1 in univariable analyses (age and number of induction courses) were included in the multivariable analysis (Table 1).

## Results

### Impact of KIR/HLA genotypes on clinical outcome

**Figure 1:**
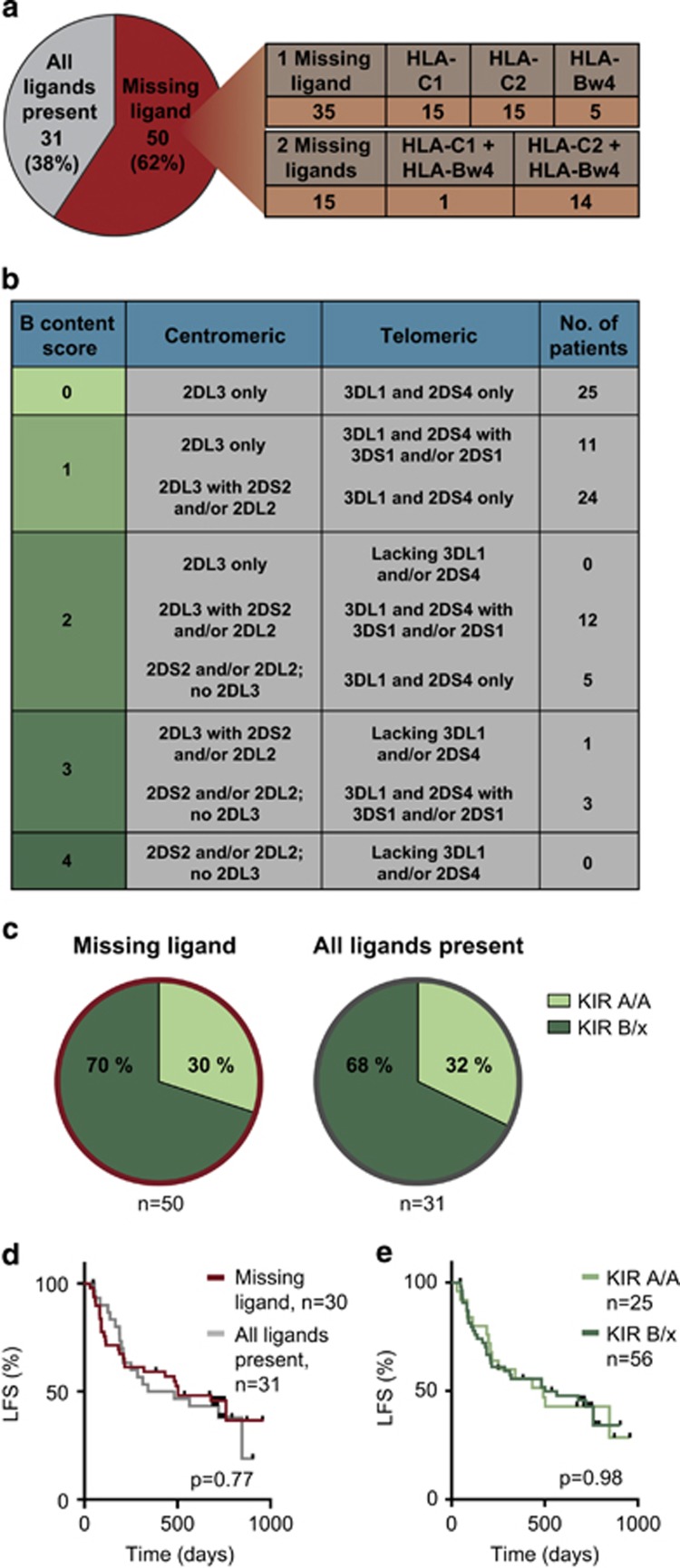
(**a**) Pie chart: number of patients with ‘all ligands present’ or ‘missing ligand’ genotypes; right: patients with the specified missing ligands. (**b**) Distribution of KIR A/A and B/x genotypes among patients within the trial. (**c**) Distribution of a KIR A/A and B/x genotype among patients with all ligands present or a missing ligand genotype. (**d**) LFS in patients with a missing ligand or all ligands present genotype. (**e**) LFS in patients with a KIR A/A or a KIR B/x genotype.

To assess the potential impact of KIR/HLA genotypes on relapse risk in patients receiving HDC/IL-2 immunotherapy, we determined the HLA and KIR genotypes in 81 AML patients in the Re:Mission trial. Thirty-one patients (38%) had a perfect match between HLA molecules and KIRs (all ligands present) and 50 patients (62%) lacked an HLA class I molecule with the cognate KIR present (missing ligand; Figure 1a). The KIR genotypes of patients were divided into A/A or B/x based on the absence or presence of haplotype B-specific genes.^10, 12, 38^ Twenty-five and 56 patients were found to be KIR A/A and KIR B/x, respectively (Figure 1b). The distribution of KIR A/A and B/x genotypes was similar between the group of patients with all ligands present and those missing one or more ligands (Figure 1c). Neither a KIR B/x genotype nor a missing ligand genotype *per se* had a significant impact on clinical outcome in terms of LFS (Figures 1d and e).

### NK cell NCR expression is clinically relevant only in patients lacking inhibitory KIR ligands or in patients with a KIR B/x genotype

**Figure 2:**
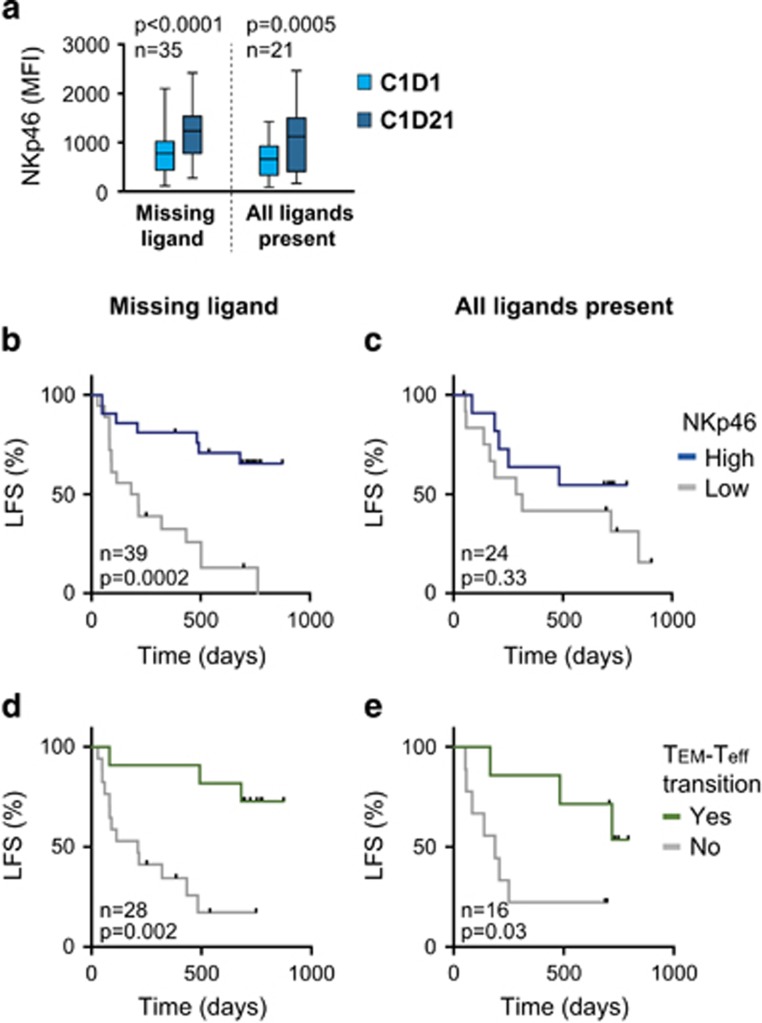
(**a**) NKp46 expression on NK cells before (C1D1) or after (C1D21) one 3-week cycle of HDC/IL-2 immunotherapy in AML patients lacking a ligand or with all ligands present (box 25–75%, whiskers min–max). (**b**, **c**) Impact of NKp46 expression on LFS. Patients were dichotomized based on above or below median expression of NKp46 on CD16^+^ NK cells on C1D21 in patients lacking a ligand (**b**) or patients with all ligands present (**c**). (**d**, **e**) Impact of CD8^+^ T-cell transition from T_EM_ to T_eff_ cells on LFS. Patients were dichotomized based on T_EM_ to T_eff_ transition or no transition of CD8^+^ T cells during the first cycle of HDC/IL-2 treatment in patients lacking a ligand (**d**) or patients with all ligands present (**e**).

Previous reports from the Re:Mission trial have shown that HDC/IL-2 immunotherapy significantly activates the NK cell compartment. NK cell killing of AML blasts *in vitro* is to a large extent dependent on intact expression of NKp46 and other NCRs.^6^ We and others have shown that expression levels of NCRs impact on relapse risk, suggesting NK cell function to be relevant to clinical outcome in AML.^5, 32, 33^ We thus sought to clarify whether the benefit of NCR expression was influenced by KIR and HLA genotypes. As shown in Figure 2, the clinical benefit of high NKp46 expression was only observed in patients with a missing ligand genotype and not in patients with all ligands present (Figures 2b and c; Table 1). The selective benefit of high NKp46 expression for patients lacking a ligand was consistently observed during therapy, that is, before treatment start (*P*=0.06 vs 0.62), at the end of the first treatment cycle (*P*=0.0002 vs 0.33), and before start of the third cycle (*P*=0.06 vs 0.41; Figure 2; Supplementary Figure 2). No differences were found regarding NCR upregulation or magnitude of NK cell expansion in blood during the first 3-week cycle of immunotherapy when comparing patients with all ligands with those with a missing ligand genotype (Figure 2; Supplementary Figure 1). In accordance with the selective benefit of NKp46 in patients with a missing ligand genotype, the benefit for older patients with above-median expression of NKp30 before treatment start^33^ was predominantly observed in patients lacking an inhibitory KIR ligand (Supplementary Figure 3). These results imply that a missing ligand genotype is needed to benefit from high NCR expression, suggesting that potentially autoreactive NS-iKIR NK cells act as effector cells in AML immunotherapy.

**Figure 3:**
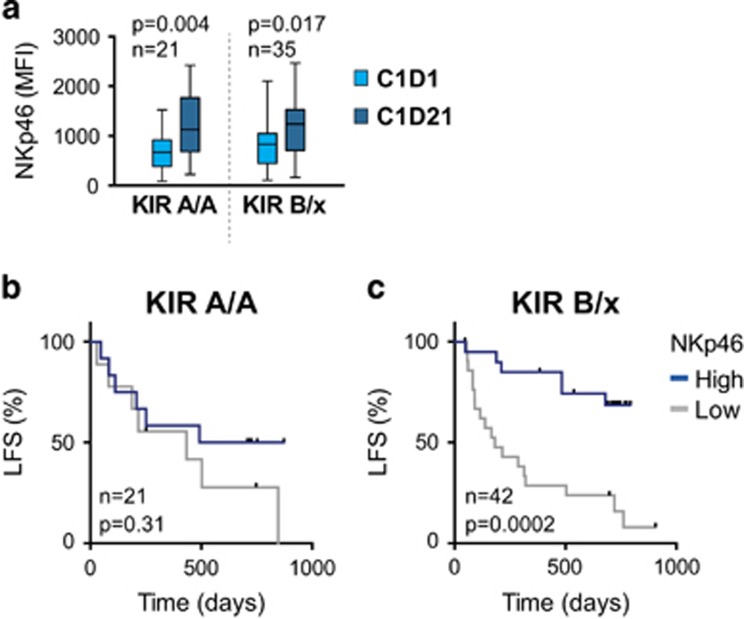
(**a**) NKp46 expression on CD16^+^ NK cells before (C1D1) or after (C1D21) one 3-week cycle of HDC/IL-2 immunotherapy in AML patients with a KIR A/A or KIR B/x genotype (box 25–75%, whiskers min–max). (**b**, **c**) Impact of NKp46 expression on LFS. Patients were dichotomized based on above or below median expression of NKp46 on CD16^+^ NK cells on C1D21 in patients with a KIR A/A (**b**) or a B/x genotype (**c**).

A KIR B/x genotype may also generate NK cell subsets that are more prone to autoreactivity. In analogy with the findings for the missing ligand genotype, the benefit of above median expression of NKp46 or NKp30 was only observed in patients with a KIR B/x genotype (*P*=0.0002 and *P*=0.003, respectively), while KIR A/A genotype patients did not benefit from high NCR expression (NKp46, *P*=0.31; Figures 3b and c, Table 1; NKp30, *P*=0.18). No differences regarding NCR upregulation or NK cell expansion were observed between patients with KIR A/A or B/x genotypes (Figure 3; Supplementary Figure 1).

### KIR/HLA genotypes prognosticate outcome independently of CD8^+^ T-cell dynamics

A recent report found that a substantial proportion of non-relapsing patients displayed a transition from CD8^+^ T effector memory cells (T_EM_) to effector cells (T_eff_) during the first 3-week-cycle of HDC/IL-2 therapy, as reflected by an increase in the frequency of T_eff_ cells along with a decrease in T_EM_ cells.^34^ We therefore determined whether the occurrence of such a CD8^+^ T-cell transition from T_EM_ to T_eff_ cells was affected by the KIR/HLA genotypes. A treatment-induced CD8^+^ T-cell transition was equally common in patients with all ligands present as compared to patients lacking a ligand, or in patients with a KIR A/A or B/x genotype (*P*=1 and 0.74; Fisher’s exact test). In contrast to NCR expression, CD8^+^ T-cell transition was associated with superior survival in patients with all ligands present and in patients lacking an inhibitory ligand (Figures 2d and e; Table 1) as well as in both KIR A/A and KIR B/x patients (Supplementary Figure 1).

### Impact of specific KIR B genes on outcome

The size of this trial prevented a thorough analysis of the individual contributions of specific activating KIR genes and their HLA ligands to the survival benefit of the KIR B/x genotype. However, among patients with above median expression of NKp46, positivity for either KIR2DS1 or KIR3DS1 was associated with improved LFS (*P*=0.04 and 0.02, respectively; Supplementary Figure 4), while KIR2DS2 and KIR2DS3 did not impact on LFS. Since the benefit of high NKp46 expression was restricted to KIR B/x and missing ligand genotypes, we next investigated the impact of having neither, one or both of these genotypes. No clear additive effect of having both KIR B/x and a missing ligand genotype was observed; however, the poor survival of KIR A/A patients with all inhibitory ligands present was evident (Supplementary Figure 4).

### Presence of NCR^+^ unlicensed NK cells in blood heralds favorable outcome

**Figure 4:**
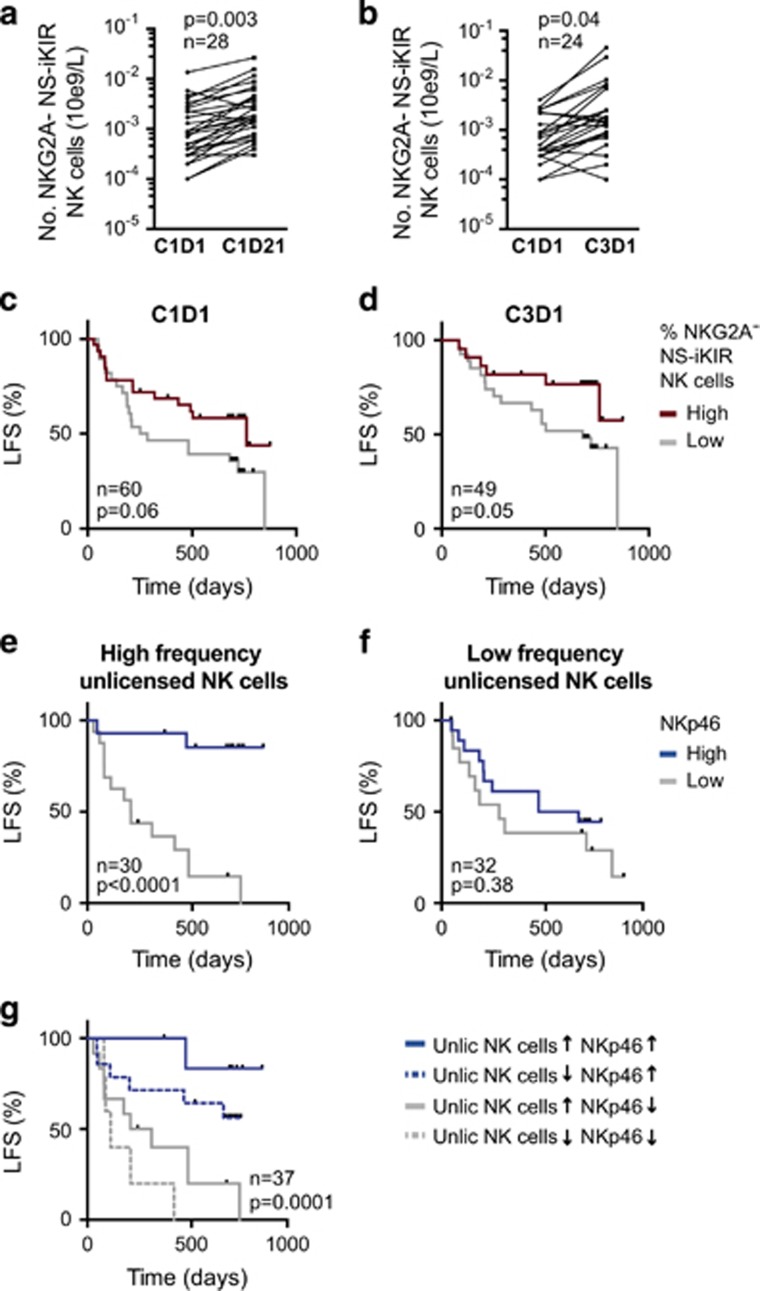
(**a**, **b**) Absolute numbers of unlicensed NKG2A^−^ NS-iKIR NK cells at indicated time points (C1D1, C1D21, C3D1) in AML patients lacking a ligand (Student’s paired *t*-test). (**c**, **d**) LFS for patients divided into high or low frequency of unlicensed NKG2A^−^ NS-iKIR NK cells at treatment start (**c**) and at start of cycle 3 (**d**). Patients were dichotomized according to receiver-operating characteristics (ROC) curves and Youden index. (**e**, **f**) Impact of NKp46 expression on LFS in patients with above or below median expression of NKp46 on CD16^+^ NK cells in patients with high (**e**) or low frequency (**f**) of NKG2A^−^ NS-iKIR NK cells. (**g**) LFS for patients lacking a ligand. Patients were divided into indicated groups based on high/low frequency of NS-iKIR NK cells and above/below median expression of NKp46. LFS was analyzed using the log-rank test (**c**–**f**) or log-rank test for trends (**g**).

Analyses of KIR/HLA genes do not provide information about the actual presence of unlicensed NK cell subsets in blood. We therefore performed KIR and CD94-NKG2A phenotyping of NK cells from patients in the Re:Mission trial. On the basis of the individual KIR/HLA genotype, the frequency and absolute counts of NK cell subsets expressing only inhibitory KIRs for non-self HLA (NS-iKIR) were determined and monitored during treatment. The frequency of NKG2A^−^ NS-iKIR NK cells varied considerably between individuals, ranging from 0.04 to 31% of all NK cells. The absolute counts of NKG2A^−^ NS-iKIR NK cells in blood followed the same pattern as previously reported for the overall population of NK cells^32^ with a strong induction of preferentially immature NK cells during a treatment cycle (Figure 4a), followed by a contraction/differentiation phase between treatment cycles. Notably, in accordance with previous results for CD56^dim^ NK cells,^32^ the number of unlicensed NKG2A^−^ NS-iKIR NK cells was significantly higher before the third treatment cycle as compared to treatment start (Figure 4b) implying that the immunotherapy may yield a sustained induction of unlicensed NK cells in blood.

Next, we determined whether high frequencies of unlicensed, NKG2A^−^ NS-iKIR NK cells impacted on clinical outcome. Patients were dichotomized to high- and low-frequency groups based on an optimal cutoff calculated using ROC curves and Youden index (cutoff 0.16 and 0.41 for C1D1 and C3D1, respectively; AUROC and CI reported in Supplementary Table 1).^37, 39^ As shown in Figures 4c and d, a high frequency of NKG2A^−^ NS-KIR NK cells before treatment start and before the third treatment cycle were associated with improved LFS (*P*=0.06 and 0.05, respectively; Table 1).

In analogy with the results achieved by comparing the outcome of patients with a missing ligand genotype or with all ligands present, the clinical benefit of high NKp46 expression was predominantly observed in patients with high frequencies of NKG2A^−^ NS-iKIR NK cells (Figures 4e and f). Notably, when segregating the patients with a missing ligand genotype into four groups based on high/low expression of NKp46 and high/low frequency of unlicensed NS-iKIR cells, we observed a pronounced additive clinical benefit of NKp46 expression and frequency of unlicensed NK cells (Figure 4g; *P*=0.0001).

### IL-2-activated NS-iKIR NK cells are reactive with primary AML blasts

**Figure 5:**
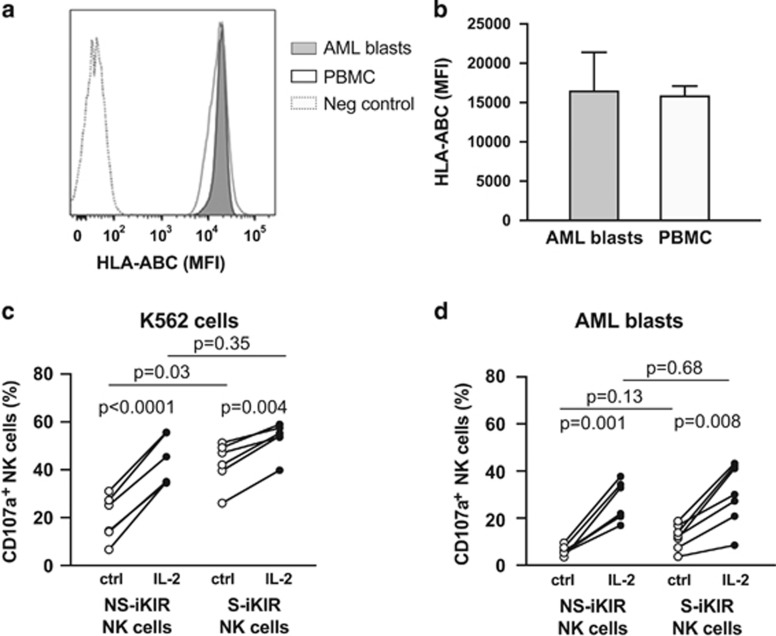
(**a**) Histogram showing HLA class I expression on CD34^+^ AML blasts and PBMCs from one representative AML patient sample. Dashed line represents unstained negative control. (**b**) Expression of HLA-ABC on leukemic 34^+^ blasts from AML patients and healthy donor PBMCs (*n*=5, *n*=3; mean + s.e.m.). (**c**, **d**) Percentage of CD107a^+^ NKG2A^−^ NS-iKIR or S-iKIR NK cells that were stimulated with 500 U/ml IL-2 overnight (filled circles) or not stimulated (ctrl; open circles) and exposed to HLA-negative K562 target cells (**c**; *n*=6) or C1/C1 matched AML blasts (**d**; *n*=7; one-way ANOVA and Bonferroni’s multiple comparison test).

The results above collectively point towards a role for unlicensed, NS-iKIR NK cells during HDC/IL-2 immunotherapy and imply that the immunotherapy may relieve the steady-state hyporesponsiveness of this NK cell subset. Previous studies have suggested that *in vitro* stimulation with inflammatory cytokines may induce responsiveness in unlicensed NK cells towards HLA-deficient target cells,^18, 28^ but few studies have compared degranulation responses of licensed vs unlicensed NK cells to primary HLA-matched leukemic cells. In a first series of experiments, we stimulated HLA-typed PBMCs from healthy donors with IL-2 and determined the degranulation responses of unlicensed or licensed, single KIR^+^ NKG2A^−^ NK cells when exposed to target cells (gating strategy in Supplementary Figure 5). In line with previous reports,^18, 25^ unstimulated NKG2A^−^ NS-iKIR^+^ NK cells responded poorly to K562 cells, while the addition of IL-2 largely abolished the difference in response to HLA-deficient target cells between NK cells expressing only NS-iKIR and NK cells expressing a single KIR specific for self-HLA (Figure 5c). As shown in Figures 5a and b, primary AML blasts generally show robust expression of HLA class I, and the relevance of HLA-deficient target cells, such as K562, to determine the anti-leukemic potential of NK cells may thus be limited. We, therefore, isolated primary CD34^+^ leukemic blasts from newly diagnosed AML patients who were homozygous for C1, and matched them with C1/C1 healthy donor NK cells in a degranulation assay. As shown in Figure 5d, while unstimulated NK cells responded poorly towards AML blasts, IL-2 stimulation significantly enhanced the response of NKG2A^−^ NS-iKIR (*P*=0.001). Notably, stimulation with IL-2 abolished the superior reactivity of licensed S-iKIR NK cells towards AML blasts, thus underscoring that unlicensed NS-iKIR NK cells may constitute an important anti-leukemic effector cell population.

## Discussion

The frequent occurrence of a genetic discordance between the set of KIR genes and the corresponding HLA genes in humans gives rise to a substantial portion of circulating NK cells that either cannot be inhibited by host HLA molecules or constantly are fed with activating signals through activating KIRs.^14, 20, 40^ Thus, to avoid attack against healthy cells, the effector function of individual NK cells is continuously adjusted, or tuned, by the input of inhibitory and activating signals from receptors binding ligands on adjacent cells.^41^ Unlicensed NK cells, which do not express inhibitory receptors for self-HLA, do not receive any inhibitory signals at steady-state and are thus hyporesponsive and display little cytotoxicity against HLA-deficient target cells.^18, 19, 42^

In this study, we aimed to clarify whether the relapse risk of AML patients receiving HDC/IL-2 immunotherapy was affected by their KIR/HLA genotypes. There was no general clinical benefit for patients with a missing ligand genotype or a KIR B/x genotype. However, it seems reasonable to assume that the NK cell population needs to be functionally intact in order to benefit from unlicensed NK cells. Previous reports from the Re:Mission trial and other studies have identified a population of patients with intact NCR expression with superior prognosis over patients with NCR expression deficiency.^5, 32, 33, 43^ A main finding in the present report was that the clinical benefit of NCR expression was strikingly restricted to patients with either a missing ligand genotype or a KIR B/x genotype. This finding suggests that HDC/IL-2 immunotherapy activates a pool of otherwise hyporesponsive, unlicensed NK cells to exert anti-leukemic activity. However, as a genetic lack of a ligand does not directly translate into a large pool of unlicensed NK cells, we used multi-color flow cytometry to monitor the presence of unlicensed NK cells during immunotherapy. In line with the observations above, we found that high frequencies of unlicensed, NKG2A^−^ NS-iKIR NK cells were associated with improved LFS. Further analyses revealed that high NKp46 expression and a high frequency of unlicensed NKG2A^−^ NS-iKIR NK cells additively improved outcome. Thus, patients with high NCR expression along with a high frequency of unlicensed NKG2A^−^ NS-iKIR NK cells displayed superior LFS. These findings suggest that functional, unlicensed NK cells may constitute an anti-leukemic effector population of relevance to the clinical outcome of HDC/IL-2 immunotherapy in AML. This hypothesis was further supported by *in vitro* experiments showing that stimulation with IL-2 triggered degranulation of unlicensed NK cells against primary AML blasts.

Naive CD8^+^ T cells differentiate via central memory and T_EM_ subsets to T_eff_ cells.^44^ We have previously reported a striking clinical benefit for patients displaying a transition from CD8^+^ T_EM_ cells to T_eff_ cells during the first cycle of immunotherapy.^34^ Multivariable analyses suggested that CD8^+^ T_EM_ to T_eff_ cell transition and high NKp46 expression independently predicted outcome (Supplementary Table 2). In contrast to the results observed for NKp46, the clinical impact of CD8^+^ T-cell transition was noted irrespective of KIR/HLA genotype, supporting that cytotoxic T cells and NK cells constitute independent effector arms that are active during immunotherapy.

The relevance of a mismatch between KIRs on NK cells and the HLA molecules present on leukemic cells for clinical outcome was first demonstrated in haploidentical allogeneic stem cell transplantation (allo-SCT), where a donor-to-recipient KIR/HLA mismatch was associated with a significant survival benefit.^45^ This finding suggests a pivotal role for alloreactive NK cells, that is, allogeneic NK cells that are licensed with respect to the donor’s set of HLA molecules but not inhibited by the recipient’s set of HLA. There is controversy regarding how long these alloreactive cells remain active: some studies support that alloreactivity will be lost as the cells adapt to the new HLA environment, while other studies imply that donor-derived hematopoietic cells may be sufficient to preserve alloreactivity, at least towards malignant cells.^46^ Moreover, even though there is no univocal conclusion concerning the role of aKIRs in leukemia, it was reported that patients receiving grafts from KIR2DS1^+^ HLA-C1/x donors had lower probability of relapse in allo-SCT of AML.^24, 47^ Our results demonstrate that the presence of unlicensed, ‘KIR/HLA mismatched’ NK cells, or the presence of aKIRs, can be clinically relevant also in non-transplanted AML patients undergoing immunotherapy. In a few other malignancies, where unlicensed NK cells have been suggested to be beneficial, immune homeostasis was disturbed by some intervention, for example, therapeutic monoclonal antibodies, cytokines or autologous transplantation.^21, 24, 25, 48^ Collectively, these studies indicate that the clinical benefit of a missing ligand genotype is related to the capacity of the therapeutic intervention to activate the unlicensed NK cell population and thereby break their tolerance to self. As these cells are more prone to mount an autoreactive response, either due to lack of inhibitory ligands or to presence of activating KIRs that are triggered by self-HLA class I, they may function as even more effective killers than licensed NK cells.^26, 48, 49^ We hypothesize that the current combination treatment, with IL-2 activating the NK cell subset and HDC targeting ROS secretion and thereby protecting the NK cells, results in an activation of otherwise hyporesponsive NK cells. The treatment is given to patients in post-consolidation phase when the leukemic burden is small. Still, it remains to be investigated if immunotherapy predominantly enables an immediate eradication of the remaining leukemic cells or if it leads to a long-lasting immune-mediated control of the malignant clone.

In conclusion, this study demonstrates that the impact of NK cells, and the predictive value of NK cell markers, during HDC/IL-2 immunotherapy is largely restricted to individuals with a missing ligand genotype and/or a KIR B/x genotype. Our results support that unlicensed, functional NK cells are relevant to prognosis in AML and that strategies to unleash the cytotoxicity of unlicensed NK cells should be considered in anti-leukemic immunotherapy.

## Figures and Tables

**Table 1:** Multivariable analyses of variables impacting on LFS

*Variable*	*Univariable analysis*			*Multivariable analysis*		
	*Hazard ratio*	*Confidence interval*	P*-value*	*Hazard ratio*	*Confidence interval*	P*-value*
NKp46 expr C1D21, pts w/ missing ligand genotype	0.207	0.083–0.516	0.001	0.219	0.087–0.554	0.001
NKp46 expr C1D21, pts w/ KIR B genotype	0.202	0.079–0.516	0.001	0.213	0.083–0.546	0.001
Transition T_EM_-T_eff_, pts w/ missing ligand genotype	0.144	0.038–0.550	0.005	0.133	0.033–0.538	0.005
Transition T_EM_-T_eff_, pts w/ all ligands present	0.208	0.042–1.025	0.054	0.075	0.009–0.626	0.017
% NKG2A^-^ NS-iKIR NK cells, C3D1, LFS	0.401	0.156–1.030	0.058	0.450	0.173–1.174	0.103
