# Supplementary material for: Impact of killer-immunoglobulin-like receptor and human leukocyte antigen genotypes on the efficacy of immunotherapy in acute myeloid leukemia
Source: Leukemia. 2017 Jun 23;31(12):2552–9. doi: 10.1038/leu.2017.151 (PMC5729331; doi:10.1038/leu.2017.151)
Supplement: Supplementary Information [file leu2017151x1.docx]

# Supplementary Information

**Supplementary Figure 1**. (**a**,**b**) Treatment-induced increase of NK cells before (circles) and after (squares) one HDC/IL-2 cycle, in AML patients with a missing ligand or with all ligands present (**a**), and in patients with a KIR A/A or B/x genotype (**b**). (Student’s paired t-test; n=30 (Missing ligand), n=17 (All ligands present), n=19 (KIR A/A), n=28 (KIR B/x)). Panels **c**-**d** show the impact of CD8^+^ T cell transition from T_EM_ to T_eff_ cells on LFS. Patients were dichotomized based on T_EM_ to T_eff_ transition (n=5 (**c**), n=12 (**d**)) or no transition (n=10 (**c**), n=16 (**d**)) of CD8^+^ T cells during the first cycle of HDC/IL-2 treatment in patients with a KIR A/A genotype (**c**) or patients with a KIR B/x genotype (**d**). LFS was compared using the logrank test.

**Supplementary Figure 2.** Impact of NKp46 expression on leukemia-free survival. Patients were dichotomized based on above or below median expression of NKp46 on CD16^+^ NK cells before the first HDC/IL-2 treatment cycle (**a**,**b**; C1D1) or at onset of the third HDC/IL-2 treatment cycle (**c**,**d**; C3D1) in patients lacking a ligand (**a**,**c)** or patients with all ligands present (**b**,**d**), (n=40 (**a**), n=22 (**b**), n=34 (**c**), n=19 (**d**)). LFS was analyzed using the logrank test.

**Supplementary Figure 3.** Impact of NKp30 expression on leukemia-free survival. Patients above 60 years were dichotomized based on above or below median expression of NKp30 on CD16^+^ NK cells before the first HDC/IL-2 treatment cycle (**a**,**b**; C1D1), after the first HDC/IL-2 treatment cycle (**c**,**d**; C1D21) or at onset of the third HDC/IL-2 treatment cycle (**e**,**f**; C3D1) in patients lacking a ligand (**a**,**c**,**e**) or patients with all ligands present (**b**,**d**,**f**); (n=22 (**a**), n=14 (**b**), n=25 (**c**), n=15 (**d**), n=20 (**e**), n=12 (**f**)). LFS was analyzed using the logrank test.

**Supplementary Figure 4.** Impact of activating KIRs and missing ligand genotype on leukemia-free survival for AML patients receiving HDC/IL-2 immunotherapy. Impact of presence or not of activating KIR2DS1 (**a**), KIR3DS1 (**b**), KIR2DS2 (**c**) or KIR2DS3 (**d**) on LFS is shown (n=32). (**e**) Above median NKp46 expression and both a KIR B/x and a missing ligand genotype (n=14), one of either KIR B/x or a missing ligand genotype (n=13), KIR A/A genotype and all ligands present (n=5), or patients with below median NKp46 expression (n=31). LFS was analyzed using the logrank test (**a**-**d**) or logrank test for trends (**e**).

**Supplementary Figure 5.** Plots show gating strategy defining single KIR2DL1^+^ or KIR2DL2/L3^+^ NKG2A^-^ NK cells.

**Supplementary Table 1**. Area under receiver-operating characteristics curve and confidence interval for receiver-operating curves (ROC) used to calculate the Youden index.

| Variable | Area under receiver-opreating characteristics curve (AUROC) | Confidence interval (CI; 95%) |
| --- | --- | --- |
| Frequency of NKG2A^-^ NS-iKIR NK cells, C1D1 | 0.586 | 0.438-0.734 |
| Frequency of NKG2A^-^ NS-iKIR NK cells, C3D1 | 0.608 | 0.445-0.770 |

**Supplementary Table 2**. Univariable and multivariable analyses of NKp46 expression and T cell transition impacting significantly on LFS.

|  | Univariable analysis | | | Multivariable analysis | | |
| --- | --- | --- | --- | --- | --- | --- |
| Variable | Hazard ratio | Confidence interval | p-value | Hazard ratio | Confidence interval | p-value |
| NKp46 expr C1D21 | 0.364 | 0.166-0.796 | 0.011 | 0.456 | 0.206-1.009 | 0.053 |
| Transition T_EM_-T_eff_ | 0.191 | 0.073-0.499 | 0.001 | 0.215 | 0.081-0.571 | 0.002 |

## Supplementary Material and Methods

### Flow cytometry of Re:Mission study samples

The following anti-human monoclonal antibodies were used for phenotyping: anti-CD3-APC-H7 (clone: SK7), CD3-FITC (HIT3a), CD4-APCH7 (RPA-T4), CD8-PerCpCy5.5 (SK1), CD14-APC-H7 (MϕP9), CD16-BV786 (3G8), CD16-V450 (3G8), CD19-APC-H7, (SJ25C1), CD56-BV711 (NCAM1), CD56-BV786 (NCAM16), CD56-APC (B159), CD45RO-PE (UCHL1), CD45RA-APC (HI100), CD107a-BV510 (H4A3), CD107a-PE-Cy7 (H4A3; all from BD Biosciences), NKG2A-PE (Z199), KIR2DL1/S1-Pe-Cy7 (EB6B), KIR2DL2/L3/S2-Pe-Cy5.5 (GL183; all from Beckman Coulter), KIR3DL1-APC (DX9), CCR7-PE-Cy7 (G043H7; both from Biolegend), NKp30-PE (AF29), NKp46-APC (9E2; both from Miltenyi Biotec), CD3-Pacific blue (S4.1, Life Technologies), KIR2DL1-AlexaFlour700 (143211; RnD Systems). Stained samples were analyzed using a 4-laser BD LSRFortessa SORP flow cytometer. Data were analyzed using the FlowJo software, version 10.1r5 (TreeStar), or BD FACSDiva Software (v.8.0.1).
